# Insulin-Like Growth Factor 1: At the Crossroads of Brain Development and Aging

**DOI:** 10.3389/fncel.2017.00014

**Published:** 2017-02-01

**Authors:** Sarah Wrigley, Donia Arafa, Daniela Tropea

**Affiliations:** ^1^School of Medicine, Trinity College DublinDublin, Ireland; ^2^Neuropsychiatric Genetics, Trinity Translational Medicine Institute St. James HospitalDublin, Ireland; ^3^Institute of Neuroscience, Trinity College DublinDublin, Ireland

**Keywords:** insulin-like growth factor 1, aging, neurodevelopment, growth factors

## Abstract

Insulin-like growth factor 1 (IGF1) is a polypeptide hormone structurally similar to insulin. It is central to the somatotropic axis, acting downstream of growth hormone (GH). It activates both the mitogen-activated protein (MAP) kinase and PI3K signaling pathways, acting in almost every tissue in the body to promote tissue growth and maturation through upregulation of anabolic processes. Overall GH and IGF1 signaling falls with age, suggesting that it is this reduced IGF1 activity that leads to age-related changes in organisms. However, mutations that reduce IGF1-signaling activity can dramatically extend the lifespan of organisms. Therefore, the role of IGF1 in the overall aging process is unclear. This review article will focus on the role of IGF1 in brain development and aging. The evidence points towards a role for IGF1 in neurodevelopment both prenatally and in the early post-natal period, and in plasticity and remodeling throughout life. This review article will then discuss the hallmarks of aging and cognitive decline associated with falls in IGF1 levels towards the end of life. Finally, the role of IGF1 will be discussed within the context of both neuropsychiatric disorders caused by impaired development of the nervous system, and neurodegenerative disorders associated with aging. IGF1 and its derivatives are shown to improve the symptoms of certain neuropsychiatric disorders caused by deranged neurodevelopment and these effects have been correlated with changes in the underlying biology in both *in vitro* and *in vivo* studies. On the other hand, studies looking at IGF1 in neurodegenerative diseases have been conflicting, supporting both a role for increased and decreased IGF1 signaling in the underlying pathogenesis of these diseases.

Insulin-like growth factor 1 (IGF1) signaling is an essential factor for early brain development, but its role in the aging brain remains unclear. In fact, while reduced IGF1 signaling with age has been historically observed as a causative factor in the aging process, correlation does not imply causation, and falling IGF1 signaling with age may in fact attenuate the effects of aging. Further work must be done in order to truly discern the role and effects of IGF1 signaling in the aging brain.

## Insulin-Like Growth Factor-1: Downstream Signaling Cascades and Cellular Effects

IGF-1 is synthesized primarily in the liver, where its synthesis is regulated by pituitary secretion of growth hormone (GH). It is central to the somatrotropic axis, acting downstream of GH to promote anabolic processes and tissue growth throughout life. IGF1 is also synthesized locally in many organs, including the brain. Both in circulation and in tissues, IGF1 is bound to high affinity IGF1-binding proteins (IGFBPs), which modulate interactions between IGF1 and its receptor.

The biological actions of IGF1 are mediated through IGF1R, a membrane-bound receptor tyrosine kinase (RTK). Binding of IGF1 to its receptor causes autophosphorylation of the intracellular component, leading to enzymatic activation and subsequent phopshorylation of the insulin receptor substrate-1 (IRS1) protein on multiple tyrosine sites. These phosphotyrosine sites then serve as docking sites for numerous intracellular signaling proteins. By bringing these interacting proteins together, complex signaling pathways are begun, including the canonical PI3-kinase and mitogen-activated protein (MAP) kinase pathways.

IGF-1R activation triggers the PI3kinase-Akt signaling pathway, which promotes cell growth and maturation. IRS1 binds PI3kinase which phosphorylates PIP2 to PIP3. PIP3 binds two protein kinases; Akt and PDK1, leading to activation of Akt, which acts on numerous proteins throughout the cell to promote cell growth and survival. Downstream substrates of Akt include mammalian target of rapamycin (mTOR), which stimulates ribosome production and protein synthesis, and Bad, a pro-apoptopic protein that is inhibited by Akt phosphorylation. Another effect of IGF1R activation through PI3kinase-Akt signaling is inhibitory phosphorylation of pro-apoptopic glycogen synthase 3β (GSK3β), which is associated with increased glycogen storage in projection neurons in the post-natal brain, as well as reduced tau hyperphosphorylation which causes neuronal death (Bondy and Cheng, 2004). IGF1-induced PI3K-Akt signaling is also linked to production of GLUT4 glucose transporters and translocation to neuronal cell membranes, promoting glucose uptake into neurons (Bondy and Cheng, 2004). This corresponds to studies demonstrating increased glucose utilization in areas of higher IGF1 and IGF1R expression in the developing brain, and reduced glucose utilization in IGF1 null brains (Cheng et al., 2000). This pathway also leads to inactivation of FOXO1, preventing FOXO-driven transcription of pro-apoptopic genes (Yin et al., 2013). Hence, PI3K signaling directly inhibits the pro-apoptopic machinery via multiple pathways.

**Figure 1:**
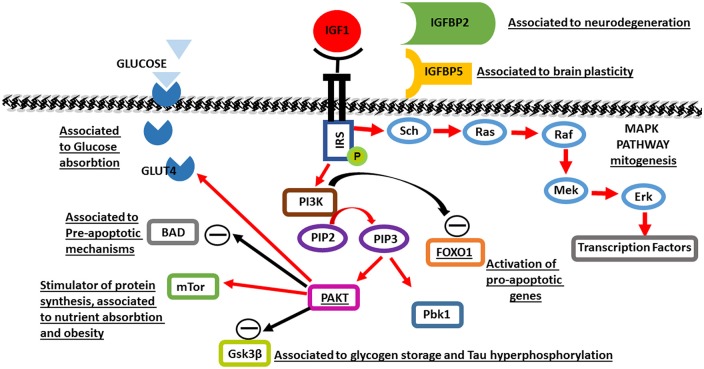
**A schematic of Insulin-like growth factor 1 (IGF1) molecular pathways activated in brain maturation and aging.** Black rows indicate inhibition, red rows indicate activation. The underlined proteins have been identified to be genetically associated to aging.

IGF1R also phosphorylates the Sch protein, which recruits the GDP2/SOS complex, leading to activation of Ras, thereby triggering the MAP kinase pathway central to growth-related gene transcription and mitogenesis (Figure 1).

## IGF1-Signaling, Aging and Lifespan in Model Organisms

IGF1 signaling is central to pathways that promote cell growth and survival, maturation and proliferation, allowing for tissue growth and renewal. Furthermore, the activity of the GH-IGF1 somatotrophic axis decreases with age, and is almost undetectable in people over 60 years (Junnila et al., 2013). This has led to many theories that upregulation of the GH/IGF1 pathway may delay aging. However, studies of IGF1 signaling on nematodes and many other species have suggested that, on the contrary, downregulation of IGF1 signaling delays aging and increases lifespan.

The IGF1 signaling pathway is an evolutionarily ancient pathway, conserved from *C. elegans* through to modern humans. The *C. elegans* IR-IGF1 receptor homolog is Daf-2. Mutations that lower the level of Daf-2 double the lifespan of the *C. elegans* model (Kenyon et al., 1993). Exactly how Daf-2 mutations increase *C. elegans* lifespan in unclear. Some daf-2 mutants adopt a quiescent state of reduced movement and fertility known as the dauer state, whereas other mutants are shown to have a lower metabolic rate, and other mutants again demonstrate a metabolic shift to fat production. However, these findings are not consistent among all daf-2 mutants, and can therefore not be coupled to lifespan extension (Kenyon, 2010). Another theory is that daf-2 mutants are better able to withstand oxidative stress (Holzenberger et al., 2003). Reduced Daf-2 activity downregulates Akt-mediated inhibition of Daf-16 (FOXO homolog), allowing Daf-16 translocation to the nucleus for target gene activation. The transcriptional targets of Daf-16/FOXO may be at least in part responsible for the stress resistance and longevity associated with Daf-2 mutants (Lin et al., 1997; Gami and Wolkow, 2006).

The impact of altered Daf-2 activity on lifespan varies between different cell lineages. While present in ectodermal, mesodermal and endodermal lineages, it is reduction in ectodermal (neuronal, skin tissue) daf-2 activity that induces a dauer state (Guarente and Kenyon, 2000). Restoration of insulin-like signaling alone in Daf-2 mutants reverts these mutants back to a wild-type lifespan, whereas restoration of insulin-like signaling in muscle or adipose tissue had no effect on lifespan (Wolkow et al., 2000). This study provides persuasive evidence that insulin/IGF1 signaling in neurons regulates lifespan. Interestingly, reduction in sensory input to the olfactory system by mutations in sensory cilia or olfactory support cell ablation can increase lifespan by up to 50% without affecting development or reproduction. This suggests that sensory neurons influence lifespan and this is at least partly mediated through Daf-2 signaling (Apfeld and Kenyon, 1999). Downstream of Daf-2 (IGF1R), mutations in age-1 (PI3K homolog) also increase lifespan, suggesting that reduction in factors downstream of IGF1 signaling are sufficient to extend lifespan (Morris et al., 1996).

The effect on lifespan by inhibition of insulin/IGF1 signaling observed in the *C. elegans* model is conserved in other species, including the *Drosophila* fly, whereby inhibiting IGF1 signaling or increasing the activity of FOXO (the Daf-16 homolog) in adipose tissue increases lifespan (Kenyon, 2010). Heterozygous mutation of the insulin receptor (IR) in *Drosophila* extends lifespan by up to 85% (Tatar et al., 2001). Furthermore, mutation of CHICO, the IRS homolog, extends lifespan by 48% in homozygotes and 36% in heterozygotes (Clancy et al., 2001). A striking inverse correlation between IGF1 levels and lifespan is also observed in mice (Kenyon, 2010). While IGF1R null mice die shortly after birth (Liu et al., 1993), heterozygous knockout (KO) of IGF1R extends lifespan by 26% compared to wild-type littermates (Holzenberger et al., 2003). This mouse model of reduced IGF1 signaling were normal in size, and displayed normal energy metabolism, but showed greater resistance to oxidative stress (Holzenberger et al., 2003).

Interestingly, this is in contrast to mice with IR or IRS KO, which show severe insulin resistance and die earlier due to hyperglycemia. From invertebrates to mammals, IGF1 signaling and insulin signaling became distinct cellular pathways with different downstream effects. Therefore, IGF1 signaling in mammals can be manipulated without interfering with systemic glucose metabolism.

In humans, lowered IGF signaling is also shown to improve longevity. Mutations known to impair IGFR function are observed in Ashkenazi Jewish centenarians (Suh et al., 2008). Additionally, Akt, FOXO3A and FOXO1A mutations are linked to longevity in numerous patient cohorts (Willcox et al., 2008; Flachsbart et al., 2009; Kenyon, 2010).

The findings that reductions in Daf-2/IGF1R signaling can radically increase lifespan would suggest that IGF1 signaling is directly linked to the aging of organisms, which is in contradiction to the theory that a fall in the activity of GH-IGF1 somatotropic axis underlies the mechanism of aging.

Although in the whole organism a general decrease of IGF signaling delays aging, this review will focus on the role of IGF1 signaling in the developing and aging brain, where IGF1 signaling in the brain promotes development and, in some cases, appears to attenuate age-related changes. Numerous studies outlined below demonstrate the neurotrophic effects of IGF1 signaling, giving evidence for promotion of neurogenesis, development and maturation, myelination, prolonged survival and resistance to injury.

## IGF1 and the Developing Brain: Expression Patterns of IGF1 and IGF1R in the Developing Brain

IGF1 is produced by all major cell types in the CNS. IGF1 expression peaks perinatally and falls throughout life, though it persists in discrete brain regions associated with continual renewal and remodeling. This is in contrast to the IGF1 receptor, which is shown to be widely expressed throughout the brain, and concentrated in neuron-rich areas including the granule cell layers of olfactory bulb, dentate gyrus and cerebellar cortex, with little hybridization in white matter regions (Bondy et al., 1992a). IGF1R is expressed in all neuroepithelial cell types, and shows a relatively stable pattern of expression from early development to maturity (Bondy et al., 1992b). There is, however, a period of increased IGF1R expression coinciding with increased IGF1 expression in specific sensory and cerebellar projecting neurons during late post-natal development (Bondy et al., 1992b). Early post-natal IGF1 expression was identified in brain regions where neurogenesis persisted after birth, including the cerebellum, olfactory bulb and hippocampus, and was shown to fall after this period of neuronal proliferation (Bach et al., 1991; Bartlett et al., 1991).

IGF1 expression persists in these areas in adult brains, but at levels much lower than that of early neonatal animals (García-Segura et al., 1991). These expression patterns highlight the association between regions of increased neurogenesis and local IGF1 and IGF1R expression during early development.

While local IGF1 expression falls shortly after birth, there exists throughout life an active transport mechanism that allows peripheral circulating IGF1 to cross the blood brain barrier (Fernandez and Torres-Alemán, 2012). This finding, combined with the fact that IGF1 receptor expression in the brain persists throughout life, suggests a role for peripherally produced IGF1 in adult brain function. Therefore, while locally produced IGF1 appears to play a role in brain function during prenatal and early post-natal development, peripherally produced IGF1 may have a continued role in the adult brain.

## IGF1 and the Developing Brain: Evidence from *In Vitro* and *In Vivo* Studies on the Effect of IGF1 on Brain Size, Neuronal Cell Number, Axonal Growth and Myelination during Early Development

IGF1 has been shown to have pleiotropic actions in all neural cells, including neurons, oligodendrocytes and astrocytes, by increasing cell number and promoting maturation and myelination. This has been proven through *in vitro* culture studies, and through both overexpression and under-expression studies in transgenic mice.

### IGF1 and the Developing Brain: Effects of IGF1 on Neural Stem Cells

*In vitro* studies examining the effect of IGF1 in neural stem cells report increased neural progenitor cell proliferation and maintenance in cell culture following treatment with IGF1 (Drago et al., 1990; Supeno et al., 2013). IGF1 was found to be more potent than insulin in stimulating mitosis of sympathetic neuroblasts (DiCicco-Bloom and Black, 1988). Furthermore, numbers of neurons produced from neural stem cell clones was increased following administration of IGF1 or heparin or withdrawal of FGF2 from cell culture, the effects of which were negated by administration of IGF1 and IGF1BP antibodies (Brooker et al., 2000). Examination of adult rat hippocampal progenitor cells showed uniform IGF1R expression, and the combined addition of IGF1 and FGF2 to these cells in culture increased DNA synthesis and cell division, without significant changes in the rate of cell death (Åberg et al., 2003).

### IGF1 and the Developing Brain: Evidence from Prenatal Overexpression and Underexpression Studies

Popken et al. (2004) demonstrated through nestin-driven overexpression of IGF1 early in the embryonic development of transgenic mice a 6% increase in brain weight by embryonic day 16, associated with a cortical plate volume 42% greater and a total cell number 54% greater in the transgenic mice compared to controls. This increase in total cell number was attributed to a 15% increase in proliferating cells in the ventricular and subventricular zones of the embryonic cerebral cortex, which give rise to the neuronal and glial cell types respectively. At post-natal day 12, a 27% increase in overall brain weight was observed, with significant increases in volume and total cell number in certain brain regions including the cerebral cortex, subcortical white matter, caudate-putamen, hippocampus, dentate gyrus and habernacular complex (Popken et al., 2004). This enhancement in neuroepithelial cell proliferation by IGF1 during embryonic neurogenesis has been shown to result from acceleration through the cell cycle (Hodge et al., 2004). Further studies in the same mouse model reported that, in ddition to enhanced proliferation of neural progenitors, there are reduced numbers of apoptopic cells throughout the cerebral cortex both prenatally and post-natally in the mice showing IGF1 overexpression, correlating with overall increased brain weight 9 months post-natally (Hodge et al., 2007).

Conversely, examination of homozygous IGF^−/−^ KO mice at 2 months demonstrates a grossly structurally normal brain, but a 38% reduction in brain weight. A reduction in parvalbumin-containing neurons in the hippocampus and striatum was shown, as well as a significant reduction in the thickness of the dentate gyrus granule cell layer (Beck et al., 1995). IGF1^−/−^ mice also show reduced dendritic length and complexity, and a 16% reduction in synaptotagmin levels, suggesting a reduction in the number of synapses, in the frontoparietal cortex (Cheng et al., 2003). A study by Liu et al. (2009) showed that brain-specific IGF1R heterozygous KO mice had brain weights 56% lower than controls at birth and 60% lower at post-natal day 90. The hippocampus was particularly affected, where the rate of growth post-natally was much lower in IGF1R heterozygous KOs compared to wild-type controls, with a greater fall in the numbers of neurons in the CA1–3 region and a smaller rise in the neuronal cell number in the dentate gyrus post-natally. This was attributed to a higher rate of cell death in IGF1R heterozygous KO mice. In all experiments performed, the phenotype was more severe in the two IGF1R homozygous KO mice that survived to adulthood, suggesting that degree of brain growth retardation was related to the level of IGF1R expression (Liu et al., 2009).

### IGF1 and the Developing Brain: Evidence from Post-Natal Overexpression and Underexpression Studies

While the above studies examined the downstream effect of prenatal IGF1 over-and under-expression on subsequent brain development, other studies had focused on the role of IGF1 in the post-natal period, given that IGF1 mRNA expression in the developing rodent brain peaks in the first two post-natal weeks of life. Using a line of transgenic mice that begin to express the transgene at birth, causing brain-restricted overexpression of IGF1, studies have shown that IGF1 overexpression causes increases in brain weight after day 10, with enlargement of the brainstem, cerebellum, cerebral cortex and hippocampus (Ye et al., 1996; Dentremont et al., 1999; O’Kusky et al., 2000). Cerebellar weight was increased by 90%, with concomitant increases in granule and Purkinje cell numbers by 82% and 20% respectively (Ye et al., 1996).

Specifically, given the persistence of IGF1 expression in the hippocampal subventricular zone and dentate gyrus post-natally, the *in vivo* actions of IGF1 on growth and development of the hippocampal dentate gyrus up to 130 post-natal days have been investigated. Transgenic IGF1 overexpression was shown to increase granule cell layer and molecular cell layer by 27%–69%, total number of neurons by 29%–61%, and total number of synapses in the molecular layer by 42%–105% compared to control littermates (O’Kusky et al., 2000). Increased neuronal numbers in the dentate gyrus in the post-natal period is thought to be due not only to increased neurogenesis but also reduced neuronal death.

Numerous studies have reported that IGF1 promotes cell survival in the post-natal brain. Transgenic mice overexpressing IGF1 have significantly fewer apoptopic cerebellar neurons at post-natal day 7 compared to wild-type controls, with increased expression of anti-apoptopic proteins and reduced expression of pro-apoptopic proteins (Chrysis et al., 2001). Similarly, in the dentate gyrus of post-natal brains, IGF1 null mice had higher rates of granule cell proliferation but lower numbers of mature granule cells, suggesting the IGF1 promotes survival of granule cells in the post-natal period (Cheng et al., 2001). For a focused review on the function of IGF1 in brain development and plasticity see Dyer et al. (2016). The role of IGF1 in improving neuronal survival and reorganization following injury in both the developing and aging brain are outlined further on in this article.

### IGF1 and the Developing Brain: Oligodendrocyte Development and Myelination

In addition to the effect of IGF1 on neuronal proliferation and survival in the developing brain, the effect on glial cells, especially oligodendrocytes, has been investigated through *in vitro* and *in vivo* studies.

*In vitro* studies show that administration of IGF1 to oligodendrocyte cells in culture promotes oligodendrocyte proliferation, differentiation, myelin production and survival (McMorris and Dubois-Dalcq, 1988; Mozell and McMorris, 1991; Barres et al., 1992; Ye and D’Ercole, 1999).

*In vivo* studies comparing transgenic mice with increased IGF-1 expression to those with IGFBP-1 expression (an inhibitor of IGF1) have shown that the mice with increased IGF-1 expression demonstrate higher numbers of oligodendrocytes, increased percentage of myelinated axons and increased thickness of myelin sheath compared to the transgenic mice with increased IGFBP-1 expression (Ye et al., 1995). In another study, prenatal overexpression of IGF1 in transgenic mice produced a mouse brain 55% larger, owing to an overall increase in total cell number, and a total myelin brain content 130% higher than that found in their non-transgenic littermates, which was not associated with a higher percentage increase in oligodendrocyte number, suggesting that increased meylin content was due to increased myelin production per oligodendrocyte (Carson et al., 1993).

Conversely, examination of IGF1 KO mice reported reduced cerebral and spinal cord white matter volume due to fewer myelinated axons and oligodendrocytes at post-natal day 55 (Beck et al., 1995). Further detailed study of IGF-1 KO mice showed consistently reduced overall brain weight 1 week post-natally compared to wild-type littermates, affecting all brain regions, as well as reduced myelination in all brain regions during the first 3 weeks of life, though this normalized and became similar to that of wild-type mice thereafter. Ths coincided with reduced levels of myelin-binding protein (MPB) and proteolipid protein (PLP) in IGF-1 KO mice during the first 3 weeks, which also normalized and became similar to controls by 10 weeks, while percentage oligodendrocyte number was persistently reduced in IGF-1 KO mice at weeks 1, 3 and 10. Additionally, reduced levels of the median subunit of neurofilament (a neuron-specific intermediate filament which acts a s a marker of axon growth) was reduced in IGF-1 KO mice and did not recover as the brain matured (Ye et al., 2002).

These studies suggest the importance of prenatal IGF1 expression in the developing brain for axon growth and CNS myelination in the early post-natal brain.

## IGF1 and the Developing Brain: Evidence from Studies of External Stimuli Known to Promote Past-Natal Cortical Maturation

Certain external stimuli have been shown to promote neurodevelopment, and in many cases these studies show that the effect is mediated by IGF1 signaling. In particular, the visual cortex is studied in the context of how brain development is affected by external sensory input. For instance, environmental enrichment (EE), defined as “a complex of inanimate and social stimulation,” is known to promote hippocampal neurogenesis and increase dendritic branching and synaptogenesis. It accelerates development of the visual cortex, causing enhanced visual acuity and increased expression of BDNF, which is known to act through the GABAergic system to promote neuroplasticity (Cancedda et al., 2004). A further study showed that this effect of EE on post-natal visual cortical development is inhibited by treatment of enriched pups with IGF1 antagonists and mimicked by treatment of non-EE pups with IGF1 infusion (Ciucci et al., 2007). Similarly, maturation of the visual system in preterm infants was accelerated by massage therapy, as evidenced both through electrophysoiogical studies and through visual acuity testing performed 3 months later. This correlated with higher serum levels of IGF1 and IGFBP3 in massaged infants. This was similarly shown in rat pups undergoing tactile stimulation, who showed faster maturation of electrophysiological tracings and higher levels of IGF1 in the brain compared to controls (Guzzetta et al., 2009).

While the above studies use the EE model to investigate the role of IGF1 in post-natal cortical maturation, other studies have used the model of monocular deprivation (MD) to assess the role of IGF1 in neuroplasticity and reorganization, whereby blocking visual input from one eye leads to structural organization of the visual cortex, allowing for ocular dominance of the intact eye. Expression of regulatory IGFBP5 gene is highly upregulated after MD, and the effects of MD on ocular dominance plasticity are negated by exogenous application of IGF1 (Tropea et al., 2006). Given that the capacity of ocular dominance plasticity in response to short deprivation is a marker of circuit immaturity, this study further supports the theory that IGF1 signaling promotes brain maturation in juvenile animals.

## IGF1 and the Adult Brain: Adult Neurogenesis

As well as the role of IGF1 in early brain development, much research has been done into the role of IGF1 in ongoing neurogenesis and CNS plasticity in the adult brain. In most regions of the brain, neurogenesis ceases after birth. IGF1 mRNA expression correlates both temporally and spatially with these periods of rapid neurogenesis in the perinatal brain. Similarly, there are certain brain regions, namely the dentate gyrus and subventricular zone of the hippocampus, where neurogenesis persists into adulthood, and this correlates with the finding that IGF1 expression in the brain is diffuse during antenatal development, but persists only in the subventricular zone and dentate gyrus of the hippocampus after birth (Anderson et al., 2002). IGF1 expression levels decrease again later in life, again at a time that corresponds with a decrease in hippocampal neurogenesis. Therefore, much research has been done to investigate how manipulation of IGF1 signaling affects adult hippocampal neurogenesis.

It has already been noted in *in vitro* studies that adult hippocampal neural progenitor cells express IGF1R, and that administration of IGF1 with FGF2 increased progenitor cell proliferation (Åberg et al., 2003). This group further suggested that low-dose IGF1 treatment triggered a small increase in the differentiation of neuronal progenitors into neurons. A further *in vivo* study used hypophysectomied rats, as this model would have low levels of circulating IGF1. Six days of peripheral subcutaneous IGF1 administration increased proliferation of neuronal progenitors in the hippocampal dentate gyrus, as evidenced by BrdU uptake. After 20 days of subcutaneous IGF1 administration, these new cells expressed neuronal-specific proteins, suggesting stimulation of neurogenesis (Aberg et al., 2000). This suggests that, while local IGF1 mRNA expression levels fall after birth, peripheral IGF1 plays a role in adult hippocampal neurogenesis.

Another study looked at the effect of local IGF1 administration on neuronal cell numbers in the dentate gyrus. Mice aged 5, 18 and 28 months were examined. The dentate gyrus was divided for analysis into the proliferative subgranular zone (PZ), the granular cell layer (GCL) and the hilus, and the numbers of new cells were quantified using BrdU labeling. This study showed that the number of BrdU labeled cells decreases with age, with 80% fewer BrdU labeled cells in the PZ of the 18 month old rats than the 5 month old rats, with a much smaller but significant decrease in BrdU labeled cells in the hilus, though no age-related changes were observed in the GCL. Intracerebroventricular infusion of IGF-I maintained an approximately three-fold increase in the number of BrdU-labeled cells in the PZ, GCL and hilus in old rats examined 31 days after BrdU injection. IGF-I did not, however, selectively induce a neuronal fate since the percentage of BrdU-labeled cells in IGF-I-treated animals that colocalized NeuN was identical to that observed in age-matched controls (Lichtenwalder et al., 2001). Transgenic overexpression of IGF1 was similarly shown to increase the proliferation of neural stem cells in the subgranular and subventricular zones of adult mice brains (Yuan et al., 2015). In this study, however, IGF1 overexpression also led to an increase in the differentiation of neuronal stem cells into neurons, in contrast to the previous study (Yuan et al., 2015).

A recent study has looked in detail at the effects of both global and brain-specific KO of IGF1 in adult hippocampal neurogenesis. Global IGF1 KO mice had both low brain IGF1 expression and low serum IGF1 levels, and showed a 2.4-fold reduction in hippocampal volume compared to controls. Using extensive immunohistochemisty studies, this group demonstrated higher staining for markers of immature differentiation from neural progenitor cells to neurons in IGF^−/−^ mice compared to controls, reduced staining for markers of the later stages of differentiation, and disorganized staining for markers of differentiated granule cells in the GCL of the dentate gyrus compared to controls (Nieto-Estévez et al., 2016b). IGF1^−/−^ cells in the GCL showed greater proliferative capacity but more immature morphology compared to controls (Nieto-Estévez et al., 2016b). The group then studied the effects of brain-specific IGF1 KO on adult hippocampal neurogenesis. These mice had normal body size and total brain volume compared to controls, but reduced volume of the GCL of the dentate gyrus. As with the global IGF1 KO mice, these mice demonstrated greater immunostaining for immature differentiation markers and more disorganized distribution of mature differentiation markers in the GCL of the dentate gyrus (Nieto-Estévez et al., 2016b). In all, this study of two IGF1^−/−^ mouse models demonstrates that a lack of IGF1 in the brain is associated with accumulation of neuronal progenitor cells, impaired transition from neural progenitor to mature granule cell neurons, a reduction in mature morphology of granule cells and disorganization of the GCL (Nieto-Estévez et al., 2016b), all of which supports the idea that IGF1 signaling is key in promoting organized adult hippocampal neurogenesis. Thus, IGF1 not only promotes adult neurogenesis through increased stem cell proliferation, but also through organized cell migration. This is similarly demonstrated in the study of IGF1 KO mice who showed reduced neuroblast migration from the subventricular zone to the olfactory bulb and poor organization of immature neurons in the olfactory bulb compared to normal mice (Hurtado-Chong et al., 2009).

Multiple processes are thought to stimulate adult neurogenesis, the best studied of which is exercise, and recent studies show that this effect is mediated through IGF1 signaling. This was shown through administration of an antibody that blocked systemic IGF1 uptake into the brain parenchyma, which reversed the exercise-induced effects on hippocampal neurogenesis (Trejo et al., 2001). Blocking IGF1R reverses exercise-induced increases in BDNF, suggesting that the downstream effects of IGF1 signaling are at least in part medicated though upregulation of brain-derived neurotrophic factor (Ding et al., 2006).

Exercise promotes functional recovery of spatial memory acquisition in rats who have undergone hippocampal injury, and attenuated the loss of motor coordination in rats following brainstem injury or Purkinje cell degeneration (Carro et al., 2001). The neuroprotective effects of exercise demonstrated in this study were reduced in rats who received subcutaneous IGF1 antibody treatment (Carro et al., 2001). Memory deficits are frequently reported in depression, and imaging studies have shown decreased hippocampal volume in patients with depression. Antidepressant therapy, including SSRIs and ECT, is known to cause upregulation of BDNF, and this has recently been shown to be IGF1-dependent (Chen and Russo-Neustadt, 2007). For a focused review on IGF1 and neurogenesis please see Nieto-Estévez et al. (2016a).

## IGF1 and the Adult Brain: Prolonged Survival, Reduced Cell Death, Resistance to Injury, Reparation and Neuroplasticity in Response to Environmental Cues

In addition to its role in neurogenesis in the adult brain, IGF1 has been studied for its effect on CNS reparation and plasticity using injury and sensory deprivation models. These studies may indicate that falling levels of IGF1 in the aging brain may indirectly lead to aging through reduced reparation, remodeling and resistance to stress.

One model of injury is the cerebellar deafferentiation model, whereby the olivocerebellar pathway is transected. The remaining olivocerebellar fibers reinnervate the hemicerebellum, but only in the early post-natal period (days 7–10). However, injection of IGF1 into the cerebellum of rats aged 11–30 days allowed for reinnervation of this pathway, indicating that this period of neuroplasticity is extended with use of IGF1 (Sherrard and Bower, 2003). IGF1-mediated reinnervation of the olivocerebellar pathway caused full motor recovery in rats rendered ataxic after 3-acetylpyridine-induced cerebellar injury (Fernandez et al., 1998).

More evidence for its role in reparative neuroplasticity is shown through the use of excitotoxicity models. Chronic intracerebral administration of IGF1 following an excitotoxic lesion in the dentate gyrus caused increased dendritic formation in young neurons in the dentate gyrus compared to untreated controls, and recovery of contextual fear memory, which is a dentate gyrus-dependent function (Liquitaya-Montiel et al., 2012). Another model of dentate gyrus injury is injection of trimethyltin, which is shown to cause elevated IGF1 mRNA levels in the hippocampus. Mice deficient in IGF1 had a significant level of CA1 hippocampal cell death, implying a role for IGF1 in CA1 hippocampal cell survival (Wine et al., 2009).

IGF1 is also thought to be protective in hypoxic-ischemic injury. Intracerebroventricular infusion of IGF1 during perinatal asphyxia in near-term foetal sheep was linked to reduced loss of striatal cholinergic and GABAergic neurons compared to controls on post-mortem examination (Guan et al., 2000). A single dose of intracerebroventricular IGF1 2 h hypoxic-ischemic injury reduced somatosensory deficits for up to 20 days after the initial insult (Guan et al., 2001). The effects of malnourishment in the post-natal period are also attenuated by IGF1 administration. Malnourished mice treated with IGF1 comparable brain weights and cell numbers compared to nourished controls, and higher numbers of oligodendrocytes and expression of myelin markers MPB and PLP, suggesting that this protective effect is through increased myelination (Ye et al., 2000).

The effects of IGF1 signaling following traumatic brain injury (TBI) have also been studied. Overexpression of IGF1 was shown to increase the density of hippocampal immature neurons following TBI. This was shown to be the result of post-traumatic proliferation and differentiation of neural stem cells into immature neurons, rather than through protection against initial insult. IGF1 overexpression also enhanced dendritic arborization of immature neurons compared to normal controls (Carlson et al., 2014). Thus, through enhanced neurogenesis and maturation, IGF1 overexpression accelerated recovery.

The role of IGF1 in activity-dependent neuroplasticity is also demonstrated through the use of sensory deprivation models. MD is a model of reduced activity-dependent activity, whereby one eye is deprived of visual stimuli, which leads to neuronal network reorganization with subsequent ocular dominance of the normal eye. IGFBP-5 is highly upregulated after MD, and administration of IGF1 promotes recovery of normal visual function in these models (Tropea et al., 2006) through upregulation of BDNF (Landi et al., 2009). Based on these findings, studies were performed to investigate whether administration of IGF1 in adult brains restores neuroplasticity. Adult rats underwent MD by eyelid suturing, and were then treated with IGF1. This was shown to trigger ocular dominance plasticity compared to MD rats untreated with IGF1 (Maya-Vetencourt et al., 2012). Furthermore, in rats rendered amblyopic by long-term sensory deprivation, those treated with IGF1 prior to restoration of sensory input showed full recovery of visual acuity compared to rats untreated with IGF1 (Maya-Vetencourt et al., 2012). Downregulation of intracortical inhibitory GABA activity, increased utilization of glucose, and interaction with 5-HT were offered as potential mechanisms through which IGF1 mediates this restoration of plasticity. An alternative sensory deprivation model is that of hindlimb unloading (HU). In adult rats, 14 days of HU decreases IGF1 levels in the somatosensory cortex (Mysoet et al., 2014) and shrinks the somatotopic representation of the hindpaw. Administration of IGF1 prevents this change in somatotopic representation (Papadakis et al., 1996; Mysoet et al., 2015). These interventional studies have demonstrated that IGF1 administration alters neuronal plasticity in response to sensory deprivation in adult animals.

The above studies outline the role of IGF1 in adult neurogenesis, reparation and reorganization in response to stressors. It is therefore possible that loss of these IGF1-driven mechanisms with age leads to age-related cognitive changes. However, numerous studies outlined later in this article report the contrary theory that it is a reduction IGF1 signaling that is neuroprotective following CNS insult.

## IGF1 and the Aging Brain: Evidence from Molecular Biology

The GH-IGF1 signaling pathway is the best characterized hormonal pathway in the process of aging. Pulsatile pituitary secretion of GH in response to stimuli such as induced hypoglycemia or arginine administration declines with age (Laron et al., 1969). This is associated with concomitant declines of circulating IGF1 levels (Johanson and Blizzard, 1981). Numerous age-related changes in the brain have been identified that suggest changes in IGF1 signaling. The density of GH receptors decreases with age, while reports are conflicting regarding age-related changes in IGF1 receptor density, with one group reporting increased density of IGF1R expression in the CA3 region of the hippocampus (Chung et al., 2002) and others reporting reduced decreased hippocampal and cortical IGF1R density in aging rats (D’Costa et al., 1993; Sonntag et al., 1999). Expression of IGF1 mRNA was reported to be reduced in the cerebellum of aging rats (Pañeda et al., 2003). These findings would suggest that IGF1 signaling is reduced in the aging brain. How this is linked to functional changes in the aging brain is unclear.

## IGF1 and the Aging Brain: Evidence from Cognitive Testing

Therefore, studies have been done to examine whether reduced IGF1 signaling is linked to cognitive dysfunction. Studies in humans found a significant correlation between better perceptual motor performance, information processing speed and fluid intelligence and higher circulating IGF1 levels (Aleman et al., 1999, 2001). Others have found a correlation between higher IGF1 levels and higher MMSE scores (Paolisso et al., 1997; Rollero et al., 1998). The MMSE is a well-validated test in terms of repeat-test reliability and tracking of cognitive function over time. In a 2 year prospective study, higher levels of circulating IGF1 levels were associated with reduced cognitive decline over the study period. However, these findings are inconsistent, with many studies also reporting no correlation between IGF1 and attention, fluid intelligence, memory or cognitive decline (Papadakis et al., 1995; Aleman et al., 1999, 2001).

## IGF1 and the Aging Brain: Evidence from Interventional Studies

In any case, correlation does not imply causation. Therefore interventional studies in human were performed to assess if GH or IGF1 levels improved cognitive function, but the results were conflicting and ultimately inconclusive (Papadakis et al., 1996; Friedlander et al., 2001). In mice, intracerebroventricular infusion of IGF1 attenuated age-related deficits in working and reference memory as assessed by Morris water maze and object recognition tasks (Markowska et al., 1998).

Further work has been done in mice studies to assess the downstream molecular effects of IGF1 administration in an aging brain. Intracerebroventricular infusion of IGF1 has been shown to increase microvascular density in aged animals (Sonntag et al., 2000b). Furthermore, it increases hippocampal NMDAR2A/B subunit expression (Sonntag et al., 2000a), which is relevant because NMDAR2B subunit ablation has been shown to impair spatial learning (Clayton et al., 2002). Local IGF1 increases local glucose utilization in the anterior cingulate cortex of aged rats (Lynch et al., 2001). Finally administration of IGF1 attenuates the age-related decline in neurogenesis in aged rats (Lichtenwalder et al., 2001).

## IGF1 and Disease: Evidence from Disorders of Neurodevelopment and Neurodegeneration

### IGF1 and Disorders of Impaired Neurodevelopment

In order to better understand the physiological roles of IGF1 in normal neurodevelopment and aging, one can look at IGF1 activity in the context of known disorders of neurodevelopment and neurodegeneration. For instance, Rett Syndrome is an X-linked neurological disorder characterized by seemingly normal post-natal development initially, followed by a sudden deterioration in function, with loss of acquired functional and motor skills at 12–18 months of age. Rather than being a neurodegenerative process, the underlying pathology is thought to be a stagnation in neuronal maturation. It is caused by a mutation in the MECP2 gene, which codes a transcriptional modulator. It is abundant in neuronal tissue and its expression correlates with that of synaptic maturation. A downstream factor of MECP2 is BDNF, which activates the same PI3K and MAPK pathways that are activated by IGF1 signaling. Therefore, subcutaneous injections if IGF1 have been administered to both mouse models and human subjects to assess if this intervention can reverse the Rett Syndrome phenotype. IGF1 has been shown to increase brain weight, dendritic spine density and levels of PSD-95, a post-synaptic scaffold protein that promotes synaptic maturation, in MECP2 null mice, as well as partially reverse the reduction in amplitude of excitatory post-synaptic current in MECP2 mice (Tropea et al., 2009). Interestingly, persistence of ocular dominance plasticity following MD, a marker of neuronal immaturity, is a feature of MECP2 mouse models of Rett syndrome. It is prevented by pre-treatment with IGF1, giving further evidence for the role of IGF1 in neuronal circuit maturation and reduction in neuroplasticity (Tropea et al., 2009; Castro et al., 2014). These studies indicate the role of IGF1 signaling in neuronal circuit maturation. Similarly, work in SHANK3 deficient models of autism have showed that treatment with IGF1 promotes maturation of excitatory synapses (Shcheglovitov et al., 2013), and reverses deficits in LTP, AMPA signaling and motor function (Bozdagi et al., 2013). Therefore, through the study of IGF1 in the context of disorders caused by poor brain development, it appears that IGF1 promotes neuronal development and brain maturation. For a review focused on IGF1 function in neurodevelopmental disorders, see Vahdatpour et al. (2016).

### IGF1 and Disorders Associated with Brain Aging

Findings through the study of IGF1 in age-related neurodegenerative disorders, however, have been contradictory, with some studies reporting that reduced IGF1 signaling is neuroprotective, while others claim that reduced IGF1 signaling with age contributes to brain aging. For instance, Alzheimer’s disease (AD) is a neurodegenerative disease associated with aging, and one group have shown that a reduction in IGF1 signaling rescues mice from AD-like pathology. An AD mouse model with reduced IGF1 signaling was created, and was reported as having reduced neuronal loss and behavioral deficits compared to the control AD mouse model with normal levels of IGF1 signaling (Cohen et al., 2009). This was attributed to tighter aggregation of Aβ plaques leading to reduced proteotoxicity. These mice also show greater resistance to oxidative stress than mice with intact IGF1 signaling (Holzenberger et al., 2003), suggesting that these findings may be due to an enhanced capacity to protect against the inflammatory effects of Aβ plaques. Similarly, another group demonstrated protection of the aging brain from amyloid pathology by knocking out neuronal IGF1R activity in the brains of adult rats (Gontier et al., 2015). In this study, IGF1R KO in adult neurons led to reduced Aβ pathology and neuroinflammation, along with preservation of spatial memory. Another study reported reduction in Aβ plaques and improved learning and memory following IRS2 KO compared to controls in APP transgenic mice (Killick et al., 2009).

This is in contrast to the prevailing theory that AD is a disorder of insulin and IGF1 resistance. AD has recently been termed “Type 3 Diabetes,” given the observation that the spectrum of Mild Cognitive Impairment—AD is associated with global reductions in glucose uptake and utilization occurring early in the course of the disease (Steen et al., 2005; Mosconi et al., 2013). In particular, hippocampal hypometabolism has been observed to correlate with faster progression to dementia (Mosconi et al., 2013). AD could therefore represent a form of CNS insulin resistance. Expression patterns of IR, IGF1 receptor (IGF1R), the intracellular substrate proteins IRS1 and IRS2, and the regulatory IGFBP-2, have been studied in brains affected by AD. One study reported increased IGF1R and decreased IGFBP-2 expression in AD brains, with higher IGF1R expression levels concentrated around amyloid plaques and in neurons with neurofibrillary tangles. These AD neurons showed decreased intracellular levels of IRS1 and IRS2, in association with greater levels of the phosphorylated inactivated forms of these proteins. These findings would suggest that AD neurons show resistance to IGF1 signaling (Moloney et al., 2010). Similarly, another study showed that cerebral neurons in AD brains demonstrate reduced responses to insulin and IGF1 signaling, mainly through phosphorylation and subsequent inactivation of IRS1 (Talbot et al., 2012). Another group that found that mutant rats with lower circulating levels of IGF1 have higher levels of Aβ plaques in the brain, and that levels of Aβ can be reduced in aging rats to levels similar to that in young rats by increasing serum levels of IGF1 (Carro et al., 2002). This is thought to be due to increased clearance of Aβ by albumin and transthyretin carrier proteins due to increased choroid plexus permeability to these proteins (Carro et al., 2002). Furthermore, IGF1R blockade in the choroid plexus worsens AD like pathology, causing amyloidosis, tau hyperphosphorylation and cognitive disturbance (Carro et al., 2006). These studies would therefore suggest that decreased insulin-IGF1 signaling in the brain at least correlates with the development of AD.

The effect of IGF1 signaling in the pathogenesis of Huntington’s disease (HD) has also been investigated. This is a triple repeat disorder caused by mutation of the HTT protein, whereby elongation of the CAG triple repeat leads to a resultant HTT protein with a prolonged polyglutamine tract. This prolongated protein is cut into toxic fragments that aggregate, causing neurotoxicity and degeneration. Reduced IGF1 signaling is linked to pathological and symptomalogical improvements in mouse models of HD. The R6/2 mouse model of HD showed more rapid neurodegeneration following increased expression of IRS2, while decreasing IRS2 expression is associated with a longer lifespan in this model (Sadagurski et al., 2011). This is attributed to fewer polyQ-HTT aggregates in the brain. Conversely, another study showed that treatment of neurons transfected with the HTT mutation with IGF1 reduced polyQ-htt aggregation through Akt-mediated huntingtin phosphorylation (Humbert et al., 2002).

## IGF1 and Metabolism: Targeting IGF1

IGF1 signaling has many effects on metabolism, at the tissue and cellular levels. This signaling is not limited to glucose and lipid homeostasis but also influences protein turnover (Sharples et al., 2015).

Manipulating IGF1 depends on our understanding of the metabolic trade-offs, especially in the brain, adipose tissue and skeletal muscle, that are associated with it. IGF and its related molecules are important in protein metabolism and the regulation of skeletal muscle mass. KO of IGFI, IGFII or the IGFI receptor causes neonatal lethality and decreases in skeletal muscle mass in rodents (Sharples et al., 2015). The role of IGF1 and its related molecules in the maintenance of skeletal muscle mass in humans is especially important for elderly individuals whose IGF1 levels decrease with age and are at risk of frailty (Maggio et al., 2013) and sarcopenia (Sharples et al., 2015). Manipulating metabolism by using dietary restriction offers a similar mechanism to reduced IGF1 signaling in that it results in the inhibition of mTOR and it has also been linked to reduced IGF1 levels. It has the potential to be used in combination with an increase in protein or amino acid intake to counteract the losses in skeletal muscle that accompany a reduction in IGF1 (Sharples et al., 2015).

Choosing which pathway to target poses an obstacle to the modification of IGF1 signaling. mTOR, which functions through complexes mTORC1 and mTORC2, might be a promising target. mTORC1 is responsive to nutrients, energy and growth factors and its inhibition has been shown to decrease aging rate and age-related weight gain in mice (Hu and Liu, 2014). KO of RAPTOR, an mTORC1-specific accessory protein, in adipose tissue preserves the lifespan extension seen in other models reducing Insulin/IGF1 signaling while also improving metabolic markers such as glucose tolerance and insulin sensitivity (Hu and Liu, 2014). This, taken with the detrimental effects of KO on tissues such as skeletal muscle and the importance of IGF1 in metabolism in the developing brain, suggests that tissue-specific reductions in signaling may be one way of overcoming this obstacle. In addition to approaches which take into account specific pathways and tissues, it may also be beneficial to investigate the differences in the effects of IGF1 reductions at different time points. Human population studies point to a positive impact of IGF1 reductions at a young age and elevations at an old age (Sharples et al., 2015).

It has been questioned whether the extended longevity afforded by reduced IGF-1 signaling and its effects on metabolism are mediated in the same way. In fact, it has recently been suggested that extended lifespan in the Ames and Snell dwarf mice is not due to IGF1 levels but rather the decrease in GH (Brown-Borg and Bartke, 2012). Furthermore, it is thought that distinct sets of neurons mediate the functions of insulin/IGF1 in the brain. KO of IRS2 in the entire brain in mice does result in lifespan extension but at 22 months these mice are also overweight, hyperinsulinemic and glucose-intolerant, demonstrating the centrality of this pathway in metabolic homeostasis. Nutrient-sensing which is central to the regulation of glucose and lipid homeostasis is carried out by the leptin-sensitive neurons of the arcuate nucleus which contain IRS2 and the IR. While it is in these neurons that insulin/IGF1 exert profound effects on metabolism, IRS2 is not required for leptin action. So then, IRS2 in these neurons mediates the functions of insulin/IGF1 ad not those of leptin, once again highlighting the importance of insulin-like signaling in metabolism. Interestingly, the decrease of IRS2 on leptin receptor-expressing neurons did not result in the increase in lifespan seen with overall IRS2 decrease. These results indicate that the neurons which act as the metabolic mediators of insulin/IGF1 signaling in the CNS are distinct from those which underlie mammalian lifespan extension due to a reduction in insulin/IGF1 signaling (Sadagurski and White, 2013; White, 2014). This provides a powerful starting point for the potential development of strategies to manipulate IGF1 function without causing metabolic dysfunction.

There is a paradox presented by the Insulin/IGF1 signaling pathway in metabolism which warrants further research. In the CNS, this paradox could well be due to the existence of neuronal subsets which mediate the effects of IGF1/Insulin and in the periphery could be owed to the differing actions of IGF1 on various tissues. The importance of IGF1 and its related molecules on metabolism is undeniable and if manipulated correctly could prove to be viable therapeutically.

## The Debate Continues

In conclusion, the above studies largely support a role for IGF1 signaling in brain development, and adult neuroplasticity and neurogenesis. However, while numerous studies report that IGF1 signaling serves to delay brain aging, and that the known fall in IGF1 signaling with age acts as a causative factor in age-related brain changes, there remain as many studies that stand in contradiction, and suggest that a reduction in IGF1 signaling delays age-related changes and diseases.

IGF1 levels are higher in the developing brain, and this is shown, through the studies outlined above, to promote neuronal development. It activates the PI3K pathway, which promotes survival by directly inactivating pro-apoptopic machinery (van der Heide et al., 2006), and increases glucose uptake by neurons (Bondy and Cheng, 2004). Post-natally, IGF1 promotes neuronal maturation, and has been shown to partially correct the phenotype of certain neurodevelopmental disorders. IGF1 is associated, in the adult brain, with regions of continued neurogenesis. These findings would suggest that IGF1 signaling exerts an overall neuroprotective effect, and that falling IGF1 levels with age contribute to the effects of aging in the brain.

However, there also exists a body of evidence suggesting that reduced IGF1 signaling attenuates the effects of aging, both in the brain and in the whole organism. A reduction in IGF1 signaling increases the life span of *C. elegans*, as DAF-2 mutants with a lower level of DAF-2 signaling have a lifespan double that of normal controls (Kenyon et al., 1993). With regards to brain function, age-related decline in axonal regeneration in *C.elegans* was shown to be regulated by DAF-2 signaling. While the rate of axonal regeneration following injury was 65% in day1 *C.elegans* worms and 28% in day 5 worms, the same experiment in DAF2^−/−^ worms had no reduction in axonal degeneration. Reduced DAF-2 signaling allowed for increased DAF-16 activity, stimulating neuronal regeneration in response to injury (Byrne et al., 2014). However, in the *C. elegans*, the insulin and IGF1 pathways are not diverged, and therefore the effects of IGF1 on aging cannot be studied in isolation. Studies of IGF1 signaling and lifespan in mammals have also been done, and an IGF1R^+/−^ transgenic mouse model with a reduced level of IGF1 signaling activity has been created that has a longer lifespan than control mice, and demonstrated greater resistance to oxidative stress (Holzenberger et al., 2003). These findings would therefore suggest that the fall in IGF1 signaling with age is not in fact the cause of aging, but is perhaps a protective mechanism that occurs as to attenuate the effects of aging.

These contradictions may arise partly because of the differential activity of IGF1 signaling in the brain compared to the whole body in different experimental models. One particular study characterizes the distinction in Ames mice, which have a primary deficiency in GH, leading to low levels of circulating IGF1. These mice demonstrate a longer lifespan, which has been attributed to absence of GH-IGF1 signaling, thereby affording evidence that IGF1 signaling contributes to aging. However, Sun et al. (2005) demonstrated that while these Ames mice show lower levels of GH and IGF1 peripherally, they have elevated levels of IGF1 in the hippocampus compared to normal mice. Furthermore, this correlated with higher levels of neurogenesis in the dentate gyrus, compared to the controls (Sun et al., 2005). This elevated level of neurogenesis in the Ames mice may underlie the observation that these mice showed less age-related cognitive deficits. Therefore, while globally reduced IGF1 signaling appears to extend the lifespan of these organisms, one should not assume that brain-specific IGF1 signaling is also reduced, or that the effect of IGF1 activity in the brain compared to the rest of the organism on the process of aging is necessarily the same.

Contradictions again arise when studying the neuroprotective effect of IGF1 signaling in hypoxic-ischemic injury. While above studies describe reduced neuronal loss following intracerebroventricular infusion of IGF1 (see above), other studies report that following Cre-LoxP-mediated inactivation of IGF1R in forebrain neurons, there was reduced neuronal damage, inflammation and edema in response to hypoxic-ischemic insult (De Maghalaes Filho et al., 2016).

These contradictions between different studies may be due to the different approaches taken: decreasing IGF1 or the receptor, or the IRS receptor, or the targeting of the modulators IGFBPs. In fact, the systems are highly regulated and the changes in each factor may have a different effect. For example, a moderate decrease in IGF1R increases life span, contrary to what happens to decreases in IGF1. Another factor to take into account is the integration insulin-IGF1 in the brain. In neurons, IRSBPs proteins and IGF1R form a complex which also binds insulin and may activate different intracellular signals. This interaction should be taken into account in therapies which use IGF1 to improve brain function, because the variability of the results may be due to different levels of insulin in the body. This theory is in line with the homeostatic function of IGF1 as a connector between body and brain. In addition, as demonstrated by Sun et al. (2005), inconsistencies also arise when the differential activity and effect of IGF1 in different organ systems is not taken into account, for it may be the case that peripheral and brain IGF1 signaling have opposing effects, with the former leading to overall acceleration of aging in the body while the latter continues to promote renewal and reparation. Furthermore, it is possible that while IGF1 signaling continues to promote neuronal development and plasticity throughout life, through its effects on cellular apoptotic machinery, glucose utilization and other neurotrophic factors, such anabolic process may simultaneously contribute to aging through accumulation of reactive oxygen species and resultant prolonged oxidative stress over time.

In all, there remains much to be done in elucidating the role of IGF1 signaling in the brain as it develops, matures and ages. IGF1 appears to act in concert with BDNF and other neurotrophic factors to promote neurogenesis and remodeling in the brain. However, its overall effects on energy metabolism and cellular oxidation may contribute to aging in all organs. What is obvious is that it is not simply a matter of high IGF1 signaling early in life promoting development and falling levels thereafter underlying the process of aging. An evolutionarily ancient pathway, IGF1 signaling has likely taken on numerous differential roles in different body tissues in health and disease (Forbes, 2016), and its complex effects on cellular maturation, tissue development and energy metabolism may contribute to organismal development and aging simultaneously.

## Author Contributions

SW wrote a consistent part of the manuscript. DA wrote part of the manuscript and contributed to the figures. DT designed the structure of the review, wrote part of the manuscript and contributed to the figures.

## Conflict of Interest Statement

DT has a patent for the potential use of IGF1 in neurodevelopmental disorders. The other authors declare that the research was conducted in the absence of any commercial or financial relationships that could be construed as a potential conflict of interest.
